# Epidemiology and Inpatient Outcomes of Invasive Aspergillosis in Patients with Liver Failure and Cirrhosis

**DOI:** 10.3390/jof11050334

**Published:** 2025-04-23

**Authors:** Aditya Sharma, Bashar Mohamad, Ayman O. Soubani

**Affiliations:** 1 Department of Internal Medicine, Wayne State University School of Medicine, Detroit, MI 48201, USA; adityamedicine@wayne.edu; 2 Division of Gastroenterology, Wayne State University School of Medicine, Detroit, MI 48201, USA; gf2633@wayne.edu; 3 Division of Pulmonary, Critical Care and Sleep Medicine, Wayne State University School of Medicine, Detroit, MI 48201, USA

**Keywords:** invasive aspergillosis, liver failure, cirrhosis, fungal infection, invasive mechanical ventilation

## Abstract

Objective: The aim of this study was to estimate the incidence and inpatient outcomes of liver failure and cirrhosis (LFC) admissions with invasive aspergillosis (IA) in the United States. Methods: This retrospective cohort study utilized the 2016–2020 National Inpatient Sample (NIS) database to analyze outcomes of IA in LFC admissions. Baseline variables, including demographics, comorbidities, and complications, were identified using International Classification of Diseases, Tenth Revision, Clinical Modification (ICD-10-CM) codes, and liver transplant admissions were excluded. Outcomes were compared between LFC admissions with and without IA. Results: During the study period, 9515 (0.36%) LFC admissions were associated with IA. This cohort experienced significantly higher rates of complications, including acute kidney injury (AKI) (73.36% vs. 42.96%; *p* < 0.001) and acute respiratory failure (ARF) (65.74% vs. 24.85%; *p* < 0.001). IA admissions required invasive mechanical ventilation (IMV) more frequently (58.17% vs. 18.78%; *p* < 0.001). All-cause inpatient mortality was significantly higher in the aspergillosis group (43.40% vs. 15.75%; *p* < 0.001). IA admissions had longer lengths of stay (LOS), with 38.89% exceeding 21 days compared to 6.20% (*p* < 0.001), and a mean LOS more than three times longer (22.9 vs. 7.5 days; *p* < 0.001). The IA group incurred over four times higher hospital charges (USD 459,414.9 vs. USD 104,389.4; *p* < 0.001) and hospitalization costs (USD 108,030.6 vs. USD 24,272.1; *p* < 0.001) compared to the LFC without aspergillosis group. Interpretation: LFC admissions with IA experienced poorer outcomes, longer hospital stays, and significantly higher healthcare costs, underscoring the need for targeted interventions in this high-risk, nonclassical population.

## 1. Introduction

Aspergillosis is a rare but fatal non-traditional infection in patients with liver failure, with previous studies reporting mortality rates exceeding 50% [1,2,3]. In recent years, there has been an increasing trend in aspergillosis infections in the United States [4]. It typically begins with inhalation of *Aspergillus* spores into the lungs, followed by hematogenous spread to other organ systems. Traditionally, fungal infections were primarily associated with severely immunocompromised individuals, such as those with hematologic malignancies, neutropenia, or T-cell suppression [5]. However, these infections are now increasingly observed in other patient populations, including those with cirrhosis [2]. Notably, approximately 50% of patients with acute or advanced liver disease are diagnosed with invasive aspergillosis postmortem [1].

Despite these alarming statistics, there remains a significant lack of data on the outcomes of LFC patients with IA. This study aims to fill this gap by providing a comprehensive overview of the epidemiology, clinical outcomes, and socioeconomic impact of IA in LFC admissions, using the NIS database from 2016 to 2020. Leveraging this extensive administrative database enables the study of rare, low-incidence conditions like IA and facilitates the calculation of nationally representative estimates.

## 2. Methods

### 2.1. Data Source

This retrospective cohort study aimed to investigate the factors associated with LFC admissions with IA and their in-hospital mortality. Additionally, the study examined the relationship between LFC admissions and other socioeconomic and hospital-level factors, such as length of stay and hospitalization cost. The study utilized the NIS database for the years 2016 to 2020, developed for the Healthcare Cost and Utilization Project, sponsored by the Agency for Healthcare Research and Quality. The NIS is a publicly available, all-payer database of inpatient hospitalizations in the United States, providing nationally representative estimates of hospital inpatient stays. It contains a stratified sample of approximately 20% of all discharges from community hospitals in the country, estimating more than 35 million hospitalizations annually. Each observation in the database represents a hospitalization with one primary diagnosis, up to 39 secondary diagnoses, and 25 procedure diagnoses [6]. These are coded using the International Classification of Diseases, Tenth Revision, Clinical Modification (ICD-10-CM) codes and the International Classification of Diseases, Tenth Revision, Procedure Coding System (ICD-10-PCS) codes. The NIS data are depersonalized, and individual identities are protected. As the study used the NIS database, it was exempt from review by the institutional review board.

### 2.2. Study Population

We analyzed the 2016–2020 NIS database to identify LFC hospitalizations using ICD-10-CM codes for acute, alcoholic, and chronic liver failure (K7200, K7201, K7040, K7041, K7210, K7211, K7290, K7291, K717, K7030, K7031, K7460, K7469). Cirrhosis was defined by combining codes for cirrhosis and complications such as portal hypertension (K766), ascites (R188), spontaneous bacterial peritonitis (K652), hepatorenal syndrome (K767), hepatic encephalopathy (K7291, K7201, K7211, K7290), and esophageal varices (I8501, I8511, I8510, I8500) [7]. Liver transplant admissions were excluded from the study and identified using ICD-10-CM (T8640, T8641, T8642, T8643, T8649, Z4823, Z944) and ICD-10-PCS (0FY00Z0, 0FY00Z1, 0FY00Z2) codes. Aspergillosis was identified using ICD-10-CM codes B440, B441, B442, B447, B448, B4481, B4489, B449, B488, and B49 listed anywhere in the diagnosis column.

### 2.3. Baseline Variables and Comparison Groups

The study evaluated baseline variables, including demographics such as age, sex, median household income, and race, categorized as White, Black, Hispanic, and Other (encompassing Asian or Pacific Islanders and Native Americans). Comorbidities, including alcohol use disorder, cerebrovascular disease, coronary artery disease, drug use disorder, dyslipidemia, obesity, and venous thromboembolism, were identified alongside complications such as AKI, ARF, disseminated intravascular coagulation, gastrointestinal bleeding, and sepsis or infection. These variables were identified using ICD-10-CM codes and the Charlson Comorbidity Index. The study also assessed the utilization of procedures such as non-invasive ventilation (NIV) and IMV and explored hospital characteristics, including teaching status, geographic region, bed size, and primary payer. A comparative analysis was conducted to identify differences in outcomes between LFC admissions with and without aspergillosis, ensuring a comprehensive evaluation of the affected population.

### 2.4. Statistical Analysis

We conducted a descriptive analysis to examine and compare these baseline variables between LFC admissions with and without IA. The provided weights were applied to generate national estimates using the methodology outlined by the Healthcare Cost and Utilization Project [8]. Categorical variables were analyzed using a paired chi-square test, while means for continuous variables were compared using the adjusted Wald test. Results were presented as percentages for categorical variables and mean ± SD for continuous variables. The ICD-10 codes utilized in the study can be found in the Supplementary Materials Table S1. Additionally, we reported annual LFC admissions from 2016 to 2020, along with the corresponding mortality for each year. Analyses were conducted using Stata software version 15.1 (StataCorp, College Station, TX, USA).

## 3. Results

### 3.1. Baseline Characteristics

From 2016 to 2020, there were 3,015,364 hospitalizations for LFC nationwide, with 9515 admissions (0.36%) associated with IA. Admissions in the IA cohort were younger compared to the non-IA cohort, with mean ages of 58.4 years and 60.3 years, respectively (*p* < 0.001). While the majority of admissions in both cohorts fell into the 61–80 age group, the IA cohort had a higher proportion of younger admissions and a lower proportion of older admissions compared to the non-IA group (18–40 years: 12.24% vs. 8.60%; 41–60 years: 39.31% vs. 40.46%; 61–80 years: 43.41% vs. 43.91%; and >80 years: 5.04% vs. 7.03%; *p* < 0.001). Although male admissions accounted for the majority in both cohorts, no significant difference was observed between the aspergillosis and non-aspergillosis groups (56.91% vs. 58.52%; *p* = 0.1632).

The majority of hospitalizations in both cohorts were White. However, the IA cohort had a significantly higher proportion of racial minorities compared to the non-IA group. Proportions of Hispanics, Blacks, and individuals from other racial groups (including Asian or Pacific Islanders and Native Americans) were higher in the aspergillosis cohort (16.12% vs. 15.49%; 13.55% vs. 10.97%; 8.62% vs. 7.08%, respectively; *p* < 0.001).

Admissions to teaching hospitals were higher for LFC patients with aspergillosis than those without aspergillosis (83.60% vs. 73.45%; *p* < 0.001). NIV and IMV were utilized more frequently in LFC admissions with IA than in those without IA (7.35% vs. 3.14% and 58% vs. 18.71%, respectively; *p* < 0.001).

The IA cohort exhibited a lower overall comorbidity burden compared to the non-aspergillosis cohort, with fewer admissions having a Charlson Comorbidity Index > 3 (57.17% vs. 61.15%; *p* < 0.001). Admissions in the IA group had significantly lower rates of alcohol use disorder (14.98% vs. 29.37%; *p* < 0.001), coronary artery disease (16.61% vs. 20.53%; *p* < 0.001), dyslipidemia (15.76% vs. 23.56%; *p* < 0.001), hypertension (45.72% vs. 57.78%; *p* < 0.001), obesity (12.14% vs. 15.04%; *p* = 0.0004), and tobacco use disorder (23.91% vs. 40.86%; *p* < 0.001). However, the IA cohort had a significantly higher rates of pulmonary comorbidities, including bronchiectasis (2.26% vs. 0.18%; *p* < 0.001) and cystic fibrosis (1.05% vs. 0.07%; *p* < 0.001). The IA group experienced a significantly higher incidence of complications compared to the non-aspergillosis LFC group. These included AKI, ARF, non-gastrointestinal bleeding, disseminated intravascular coagulation, intracranial hemorrhage, sepsis, and venous thromboembolism (Table 1).

### 3.2. Outcomes

All-cause inpatient mortality was notably higher in the IA group (43.40% vs. 15.75%; *p* < 0.001). Additionally, nearly half of IA admissions required discharge to a nursing or rehabilitation facility (45.21%) compared to 18.58% in the non-aspergillosis group, while routine discharges were significantly more common in the non-aspergillosis group (24.93% vs. 5.98%; *p* < 0.001).

**Figure 1:**
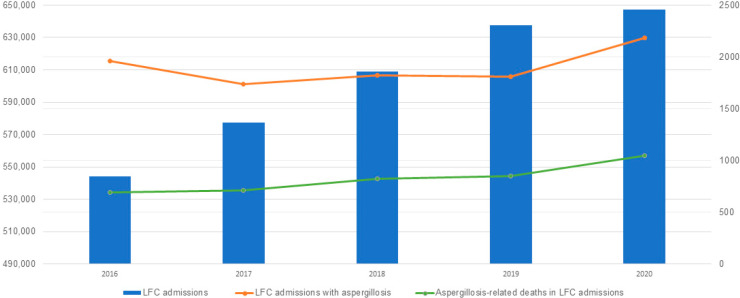
Liver Failure and Cirrhosis Admissions in the US (2016–2020).

Hospital stays were markedly longer for the IA cohort, with 38.89% of admissions exceeding 21 days compared to 6.20% in the non-aspergillosis cohort (*p* < 0.001). The mean LOS was more than three times longer for IA admissions (22.9 vs. 7.5 days; *p* < 0.001). The mean total hospital charges for the IA cohort were more than four times higher than those for the non-aspergillosis cohort (USD 459,414.9 vs. USD 104,389.4; *p* < 0.001). Similarly, the mean total hospitalization costs in the IA group were over four times greater than in the non-IA group (USD 108,030.6 vs. USD 24,272.1; *p* < 0.001) (Table 1). Table 2 and Figure 1 provide the number of admissions and death of LFC admissions with IA during the study period.

## 4. Discussion

To our knowledge, this is the largest study utilizing a national database to examine the epidemiology and outcomes of IA in patients with liver disease. Fungal infections have long been linked to severe immunosuppression, commonly seen in conditions such as hematologic malignancies, neutropenia, and T-cell suppression [5]. While aspergillosis has traditionally been recognized as an opportunistic infection, recent studies have highlighted its role as a life-threatening complication in patients with liver failure [1,9,10]. A previous study suggests that the increased infection risk in cirrhotic patients may be due to cirrhosis-associated immune dysfunction, which combines immunodeficiency with disturbances in key immune cells, including neutrophils, monocytes, T cells, and B cells [11]. Further supporting this, another study found that both innate and, more notably, adaptive cellular immune impairments emerge early in the progression of liver cirrhosis, potentially increasing susceptibility to infections [12]. A postmortem study revealed that over half of the patients with acute or advanced liver disease had a diagnosis of invasive aspergillosis [1]. The Centers for Disease Control and Prevention data indicate that the incidence of aspergillosis has been rising at an average rate of 3% over the past decade [13]. Given the increasing incidence of aspergillosis and the growing recognition of LFC patients as a high-risk group, our study seeks to address gaps in epidemiological data regarding its prevalence, outcomes, and clinical implications in this vulnerable population.

Between 2016 and 2020, we identified 9515 hospitalizations for LFC and IA, corresponding to an observed incidence of 0.36%. This is similar to findings from a retrospective study in China, which reported an incidence of 0.29% [14]. However, significantly higher rates have been observed in intensive care unit (ICU) settings, reaching 14.28% [10]. Prior research has classified liver cirrhosis patients with an ICU stay of more than 7 days as an intermediate-risk category for invasive aspergillosis, possibly explaining the higher incidence observed in ICUs [2]. Notably, liver transplant recipients have a significantly higher incidence of aspergillosis, exceeding 9.2% [15]. Solid organ transplantation is a well-recognized risk factor for opportunistic aspergillosis, primarily due to prolonged immunosuppressant and corticosteroid exposure. To ensure a more specific analysis, we excluded hospitalizations involving liver transplantation from this study.

The mean age of LFC admissions with IA in our study was 58.4 years, closely aligning with a previous study that reported a mean age of 57 years for LFC patients [16]. We observed a higher incidence of IA among African-American, Hispanic, Asian, Pacific Islander, and other minority populations. Among these groups, Hispanics had the highest proportion of aspergillosis cases (16.27%), underscoring the disproportionately higher burden of aspergillosis in minority communities affected by LFC. This trend is consistent with prior research on COVID-19-associated aspergillosis [17]. Furthermore, LFC admissions with IA were more frequently seen in teaching hospitals compared to those without aspergillosis (83.39% vs. 73.36%). This pattern mirrors findings from a study on influenza-associated aspergillosis and is likely due to the transfer of more critically ill patients—who have a higher risk of developing IA—to academic centers, as well as greater awareness and diagnostic recognition of aspergillosis in teaching hospitals [18].

Our study found that admissions with IA had a younger mean age at admission and a lower Charlson Comorbidity Index (19.18% vs. 29.14% with Charlson Comorbidity Index > 3; *p* < 0.001), suggesting that IA is more closely linked to acute physiological decline rather than cumulative comorbidities. This aligns with the nature of infections, which typically occur as acute events rather than as a consequence of long-term disease burden.

We observed a higher proportion of IA-associated LFC admissions in the wealthiest income quartile, whereas the poorest quartile showed a lower proportion of LFC admissions with IA. This disparity may reflect greater access to healthcare resources and diagnostic tools among higher-income populations, potentially leading to increased detection of IA. However, the database lacks granular information to provide definitive explanation of this observation.

We found that frequency of AKI was significantly higher in the IA cohort compared to the non-IA cohort (73.36% vs. 42.96%). Previous studies have similarly reported that IA contributes to severe organ failure [18,19]. Additionally, we observed a higher incidence of non-gastrointestinal bleeding, disseminated intravascular coagulation, intracranial hemorrhage, and venous thromboembolism in the IA cohort. These complications indicate an increased risk profile that may hinder the feasibility of invasive diagnostic procedures, potentially leading to delays in diagnosis and poorer clinical outcomes. We observed that ARF occurred more than twice as frequently in the IA cohort, a trend consistent with previous studies on IA [18]. This reflects the more critical nature of patients with IA or may be a reason for their increased risk for this infection. Additionally, pulmonary comorbidities such as cystic fibrosis and bronchiectasis were more prevalent in this group. Individuals with underlying lung disease are known to be at increased risk for IA, which typically originates in the lungs and can disseminate to other organs. LFC admissions with IA required IMV over three times more frequently than those without aspergillosis (58.17% vs. 18.78%). Western European studies have reported IMV requirements ranging from 52% to 100% in cirrhotic patients with aspergillosis [10,16]. This high IMV requirement may be due to missed or delayed diagnosis of IA in these non-classical patients, a pattern also observed in previous studies. Diagnostic tools for aspergillosis in non-neutropenic patients, such as serum galactomannan, have limited predictive value, whereas bronchoalveolar lavage specimens have demonstrated greater diagnostic accuracy in this population [9,20]. A recent study highlighted the potential of a non-invasive antibody-guided positron emission tomography and magnetic resonance probe for detecting *Aspergillus fumigatus* lung infections [21]. Given these challenges, diagnosing aspergillosis in non-neutropenic patients requires a comprehensive approach that integrates radiological findings, cultures, fungal biomarkers, and molecular tools for greater accuracy, as also recommended in the structured diagnostic and therapeutic framework proposed by Bassetti et al. [9].

The length of stay for admissions with IA was more than three times longer than that of non-aspergillosis patients (22.9 vs. 7.5 days) and significantly exceeded durations reported in previous large-scale studies, which documented ICU stays of 14 or more days for aspergillosis patients [2,10,22]. The extended hospitalization can likewise be attributed to the atypical presentation and delayed diagnosis of IA in LFC patients.

We found that the mean total hospital charges for the IA cohort were more than four times higher than those for the non-IA cohort (USD 459,414.9 vs. USD 104,389.4). Similarly, mean total hospitalization costs were over four times greater in the aspergillosis group compared to the non-aspergillosis group (USD 108,030.6 vs. USD 24,272.1), placing a significant financial strain on the healthcare system. An interesting observation from a study in China highlighted that the high mortality associated with invasive pulmonary aspergillosis was not only due to diagnostic and treatment challenges but also to the heavy economic burden. Many patients, unable to afford the cost of care, ultimately discontinued treatment [3]. These findings emphasize the critical need for early diagnosis, improved treatment strategies, and cost-effective interventions to reduce both clinical and financial burdens.

Mortality rates for aspergillosis in cirrhotic patients have remained alarmingly high, with prior studies reporting rates exceeding 50% [1,2,3]. A cohort study from China observed mortality surpassing 70%, while a decade-long analysis from France documented a lower hospital mortality rate of 37% [3,16]. Additionally, a large-scale study on ICU patients with cirrhosis and aspergillosis has reported a 100% mortality rate [10]. In our study, we found a mortality rate of 43%, aligning more closely with the French data. Compared to neutropenic patients, non-neutropenic individuals are significantly less likely to exhibit classic symptoms of IA, making early detection more challenging [23]. Furthermore, a large study examining both neutropenic and non-neutropenic patients found that classic radiographic signs, such as nodules and cavitation, were rare. Instead, non-specific findings like consolidation, ground-glass infiltrates, and pleural effusions were more commonly observed [23]. This lack of distinct imaging features further complicates the diagnosis of aspergillosis in LFC patients. The combination of delayed diagnosis and atypical presentation often leads to aspergillosis being recognized only at critical stages, contributing to poorer outcomes. Another important factor that may play a role in the outcome of aspergillosis in this patient population is the challenge with starting and maintaining antifungal therapy given the increased risk of liver dysfunction associated with these medications. The NIS database does not provide information about antifungal therapy in these patients.

To our knowledge, this is the largest study examining aspergillosis as a complication in liver failure patients. It provides valuable insights into its incidence and outcomes; however, several limitations must be acknowledged.

One of the limitations is the lack of data on diagnostic tools and treatment strategies, as the NIS database does not capture this information. Specifically, the database does not provide information on the etiology of LFC, treatment such corticosteroids or other immunomodulatory medications, or antifungal therapy. Additionally, the structure of the NIS prevents tracking individual patients across multiple admissions, with each entry representing a separate hospitalization rather than a continuous patient history. This restriction limits longitudinal analysis and may obscure disease progression and treatment pathways [24]. Furthermore, the NIS lacks the clinical information needed to apply the European Organization for Research and Treatment of Cancer criteria for IA, including host factors indicative of immunocompromised status. Given the low observed incidence and the inability to exclude alternative diagnoses such as chronic pulmonary aspergillosis or to identify established host risk factors, establishing a definitive association or causal relationship between LFC and IA is challenging. Moreover, since the timing of diagnoses cannot be determined in the NIS, it remains unclear whether IA represents a pre-existing condition, the cause of admission, or a complication arising during hospitalization.

While national databases offer valuable epidemiological insights, findings should be interpreted cautiously due to the risk of misclassification bias and inconsistencies from coding errors and variations in documentation practices across healthcare facilities. Given these limitations, generalizing results from database studies requires careful consideration.

This study, the largest to date, highlights the significant burden of IA in LFC patients, who experience worse clinical outcomes, prolonged hospital stays, and higher healthcare costs. Diagnosing invasive aspergillosis remains challenging due to lack of awareness, the atypical presentation, and the limited sensitivity and specificity of diagnostic studies. A comprehensive diagnostic approach integrating imaging, cultures, fungal biomarkers, and molecular tools is crucial for early detection and timely intervention.

These findings underscore the urgent need for targeted interventions, interdisciplinary collaboration, and cost-effective strategies to improve outcomes in this high-risk yet underrecognized population. Strengthening diagnostic accuracy and optimizing treatment approaches are essential to reducing both clinical and economic burdens.

## Figures and Tables

**Table 1:** Baseline Variables of Admissions with Liver Failure and Cirrhosis With and Without Aspergillosis.

Variable	LFC Without Aspergillosis (N: 3,005,849)	LFC with Aspergillosis (N: 9515)	*p * Value
Age at admission in years, mean (±SD)	60.3 (13.5)	58.4 (14.3)	<0.001
Age group (in years):			<0.001
18–40	8.60%	12.24%	
41–60	40.46%	39.31%	
61–80	43.91%	43.41%	
>80	7.03%	5.04%	
Race:			0.0001
White	66.48%	61.66%	
Black	10.97%	13.58%	
Hispanic	15.47%	16.27%	
Others ^a^	7.08%	8.49%	
Female	41.48%	43.09%	0.1632
Quarter:			0.1219
Quarter 1 (January–March)	24.93%	25.43%	
Quarter 2 (April–June)	24.36%	24.12%	
Quarter 3 (July–September)	25.11%	23.06%	
Quarter 4 (October–December)	25.61%	27.38%	
Median household income:			0.0036
Quartile 1 (poorest)	33.07%	30.77%	
Quartile 2	26.93%	26.14%	
Quartile 3	23.07%	23.02%	
Quartile 4 (wealthiest)	16.93%	20.06%	
Primary expected payer:			<0.001
Medicare	47.51%	45.22%	
Medicaid	23.31%	22.98%	
Private Insurance	20.45%	24.87%	
Self-pay and other ^b^	8.73%	6.94%	
Admitted to teaching hospital	73.36%	83.39%	<0.001
Hospital bed size:			<0.001
Small	17.89%	12.45%	
Medium	28.00%	23.33%	
Large	54.11%	64.21%	
Region of Hospital:			0.0565
Northeast	16.42%	17.66%	
Midwest	20.58%	19.39%	
South	39.77%	37.52%	
West	23.22%	25.43%	
Charlson comorbidity index:			<0.001
0–1	8.77%	13.45%	
2–3	30.08%	29.37%	
>3	61.15%	57.17%	
Comorbidities:			
Alcohol use disorder	29.37%	14.98%	<0.001
Bronchiectasis	0.18%	2.26%	<0.001
Cerebrovascular disease	4.66%	10.14%	<0.001
Coronary artery disease	20.53%	16.61%	<0.001
Cystic fibrosis	0.07%	1.05%	<0.001
Drug use disorder	8.79%	7.04%	0.0072
Dyslipidemia	23.56%	15.76%	<0.001
Hypertension	57.78%	45.72%	<0.001
Obesity	15.04%	12.14%	0.0004
Tobacco use disorder	40.86%	23.91%	<0.001
Complications:			
Acute kidney injury	42.96%	73.36%	<0.001
Acute respiratory failure	24.85%	65.74%	<0.001
Bleeding other than GI	1.27%	4.15%	<0.001
Disseminated intravascular coagulation	2.49%	11.46%	<0.001
Gastrointestinal bleeding	20.49%	21.97%	0.1147
Intracranial hemorrhage	1.42%	3.52%	<0.001
Sepsis or infection	52.95%	100.00%	<0.001
Venous thromboembolism ^c^	7.99%	16.29%	<0.001
Procedure:			
Invasive mechanical ventilation	18.78%	58.17%	<0.001
Non-invasive ventilation	3.15%	7.30%	<0.001
Length of stay (in days):			<0.001
≤10	80.05%	33.32%	
11–20	13.75%	27.80%	
≥21	6.20%	38.89%	
Disposition of patient:			<0.001
Routine	50.98%	24.93%	
Transfer to short-term hospital	4.93%	7.26%	
Transfer to a facility ^d^	22.94%	45.21%	
Home-health care	18.58%	21.12%	
Left against medical advice	2.57%	1.49%	
Outcomes:			
All-cause inpatient mortality	15.75%	43.40%	<0.001
Length of stay in days, mean (±SD)	7.5 (9.4)	22.9 (24.7)	<0.001
Total hospital charges in US dollars, mean (±SD)	$104,389.4 (200,464.1)	$459,414.9 (682,279.8)	<0.001
Total hospital costs in US dollars, mean (±SD)	$24,272.1 (43,016.1)	$108,030.6 (155,198.1)	<0.001

^a^: Includes Asian or Pacific Islanders, Native Americans, and others. ^b^: Includes no charge, worker’s compensation, CHAMPUS, CHAMPVA, Title V, and other government programs. ^c^: Includes both pulmonary embolism and deep vein thrombosis. ^d^: Includes Skilled Nursing Facility (SNF), Intermediate Care Facility (ICF), another type of facility.

**Table 2:** Absolute Number of Liver Failure and Cirrhosis Admissions (2016–2020).

	2016	2017	2018	2019	2020	Total
Admissions for liver failure and cirrhosis	544,150	577,480	608,745	637,660	647,330	3,015,364
Admissions for liver failure and cirrhosis with invasive aspergillosis	1960	1740	1820	1810	2185	9515
Death count in admissions for liver failure and cirrhosis with invasive aspergillosis	695	710	825	850	1045	4125

## Data Availability

The original contributions presented in the study are included in the article/Supplementary Materials, further inquiries can be directed to the corresponding author.

## Supplementary Materials

The following supporting information can be downloaded at: https://www.mdpi.com/article/10.3390/jof11050334/s1, Table S1: The quartile classification of the estimated median household income of residents in the patient’s ZIP code vary by every year. Listed below are the dollar ranges for study duration 2017–2019.

## Abbreviations

AKI	Acute kidney injury
ARF	Acute respiratory failure
IA	Invasive aspergillosis
ICD-10-CM	International Classification of Disease, Tenth Revision, Clinical Modification
ICD-10-PCS	International Classification of Diseases, Tenth Revision, Procedure Coding System
ICU	Intensive care unit
IMV	Invasive mechanical ventilation
LFC	Liver failure and cirrhosis
LOS	Length of stay
NIS	National inpatient sample
NIV	Non-invasive ventilation
SD	Standard deviation
