# Supplementary material for: Epidemiology and Inpatient Outcomes of Invasive Aspergillosis in Patients with Liver Failure and Cirrhosis
Source: J Fungi (Basel). 2025 Apr 23;11(5):334. doi: 10.3390/jof11050334 (PMC12112825; doi:10.3390/jof11050334)
Supplement: Supplementary file 1 [file jof-11-00334-s001.zip › jof-3564453-supplementary.pdf]

Supplementary Materials:

A: Diagnosis codes {International Classification of Diseases, Tenth Revision Clinical Modification (ICD-10-CM)}

1. Acute kidney injury: N170, N171, N172, N178, N179
2. Acute liver failure: K7200, K7201
3. Acute respiratory failure: J80, J95821, J95822, J9600, J9601, J9602, J9620, J9621, J9622, R092
4. Alcohol use disorder: F10*
5. Alcoholic hepatic failure: K7040, K7041
6. Ascites: R188
7. Aspergillosis: B440, B441, B442, B447, B448, B4481, B4489, B449, B488, B49
8. Bleeding other than gastrointestinal: N938, N939, N950, D7801, D7802, D7821, D7822, E3601, E3602, E89810, E89811, G9731, G9732, G9751, G9752, H05231, H05232, H05233, H05239, H31301, H31302, H31303, H31309, H31311, H31312, H31313, H31319, H3560, H3561, H3562, H3563, H4310, H4311, H4312, H4313, H47021, H47022, H47023, H47029, H59111, H59112, H59113, H59119, H59121, H59122, H59123, H59129, H59311, H59312, H59313, H59319, H59321, H59322, H59323, H59329, H9521, H9522, H9541, H9542, I97410, I97411, I97418, I9742, I97610, I97611, I97618, I9762, I97620, J9501, J9561, J9562, J95830, J95831, L7601, L7602, L7621, L7622, M96810, M96811, M96830, M96831, N421, N99510, N99520, N99530, N9961, N9962, N99820, N99821, O031, O036, O046, O071, O081, O208, O209, O4410, O4410, O4411, O4411, O4412, O4412, O4413, O4413, O4430, O4430, O4431, O4431, O4432, O4432, O4433, O4433, O4450, O4450, O4451, O4451, O4452, O4452, O4453, O4453, O46001, O46002, O46003, O46009, O46011, O46012, O46013, O46019, O46021, O46022, O46023, O46029, O46091, O46092, O46093, O46099, O468X1, O468X2, O468X3, O468X9, O4690, O4691, O4692, O4693, O670, O670, O678, O678, O679, O679, O720, O720, O721, O721, O722, O722, R041, R0489, R049, R58, T792XXA, T82837A, T82838A, T8383XA, T8483XA, T85830A, T85838A, T8583XA, O26851, O26852, O26853, O26859
9. Bronchiectasis: J470, J471, J479
10. Cerebrovascular disease: G43601, G43609, G43611, G43619, I6000, I6001, I6002, I6010, I6011, I6012, I602, I6020, I6021, I6022, I6030, I6031, I6032, I604, I6050, I6051, I6052, I606, I607, I608, I609, I610, I611, I612, I613, I614, I615, I616, I618, I619, I6200, I6201, I6202, I6203, I621, I629, I6300, I63011, I63012, I63013, I63019, I6302, I63031, I63032, I63033, I63039, I6309, I6310, I63111, I63112, I63113, I63119, I6312, I63131, I63132, I63133, I63139, I6319, I6320, I63211, I63212, I63213, I63219, I6322, I63231, I63232, I63233, I63239, I6329, I6330, I63311, I63312, I63313, I63319, I63321, I63322, I63323, I63329, I63331, I63332, I63333, I63339, I63341, I63342, I63343, I63349, I6339, I6340, I63411, I63412, I63413, I63419, I63421, I63422, I63423, I63429, I63431, I63432, I63433, I63439, I63441, I63442, I63443, I63449, I6349, I6350, I63511, I63512, I63513, I63519, I63521, I63522, I63523, I63529, I63531, I63532, I63533, I63539, I63541, I63542, I63543, I63549, I6359, I636, I638, I639, I6601, I6602, I6603, I6609, I6611, I6612, I6613, I6619, I6621, I6622, I6623, I6629, I663, I668, I669, R29700, R29701, R29702, R29703, R29704, R29705, R29706, R29707, R29708, R29709, R29710, R29711, R29712, R29713, R29714, R29715, R29716, R29717, R29718, R29719, R29720, R29721, R29722, R29723, R29724, R29725, R29726, R29727, R29728, R29729, R29730, R29731, R29732, R29733, R29734, R29735, R29736, R29737,

R29738, R29739, R29740, R29741, R29742, I6501, I6502, I6503, I6509, I651, I6521, I6522, I6523, I6529, I658, I659, G460, G461, G462, G463, G464, G465, G466, G467, G468, I671, I672, I675, I676, I677, I6781, I6782, I6783, I67841, I67848, I6789, I679, I680, I682, I688, G450, G451, G452, G453, G454, G458, G459, I6900, I6901, I69010, I69011, I69012, I69013, I69014, I69015, I69018, I69019, I69020, I69021, I69022, I69023, I69028, I69031, I69032, I69033, I69034, I69039, I69041, I69042, I69043, I69044, I69049, I69051, I69052, I69053, I69054, I69059, I69061, I69062, I69063, I69064, I69065, I69069, I69090, I69091, I69092, I69093, I69098, I6910, I6911, I69110, I69111, I69112, I69113, I69114, I69115, I69118, I69119, I69120, I69121, I69122, I69123, I69128, I69131, I69132, I69133, I69134, I69139, I69141, I69142, I69143, I69144, I69149, I69151, I69152, I69153, I69154, I69159, I69161, I69162, I69163, I69164, I69165, I69169, I69190, I69191, I69192, I69193, I69198, I6920, I6921, I69210, I69211, I69212, I69213, I69214, I69215, I69218, I69219, I69220, I69221, I69222, I69223, I69228, I69231, I69232, I69233, I69234, I69239, I69241, I69242, I69243, I69244, I69249, I69251, I69252, I69253, I69254, I69259, I69261, I69262, I69263, I69264, I69265, I69269, I69290, I69291, I69292, I69293, I69298, I6930, I6931, I69310, I69311, I69312, I69313, I69314, I69315, I69318, I69319, I69320, I69321, I69322, I69323, I69328, I69331, I69332, I69333, I69334, I69339, I69341, I69342, I69343, I69344, I69349, I69351, I69352, I69353, I69354, I69359, I69361, I69362, I69363, I69364, I69365, I69369, I69390, I69391, I69392, I69393, I69398, I6980, I6981, I69810, I69811, I69812, I69813, I69814, I69815, I69818, I69819, I69820, I69821, I69822, I69823, I69828, I69831, I69832, I69833, I69834, I69839, I69841, I69842, I69843, I69844, I69849, I69851, I69852, I69853, I69854, I69859, I69861, I69862, I69863, I69864, I69865, I69869, I69890, I69891, I69892, I69893, I69898, I6990, I6991, I69910, I69911, I69912, I69913, I69914, I69915, I69918, I69919, I69920, I69921, I69922, I69923, I69928, I69931, I69932, I69933, I69934, I69939, I69941, I69942, I69943, I69944, I69949, I69951, I69952, I69953, I69954, I69959, I69961, I69962, I69963, I69964, I69965, I69969, I69990, I69991, I69992, I69993, I69998

11. Chronic hepatic failure: K7210, K7211, K7211
12. Coronary artery disease : I200, I201, I208, I209, I240, I248, I249, I2510, I25110, I25111, I25118, I25119, I252, I255, I256, I25700, I25701, I25708, I25709, I25710, I25711, I25718, I25719, I25720, I25721, I25728, I25729, I25730, I25731, I25738, I25739, I25750, I25751, I25758, I25759, I25760, I25761, I25768, I25769, I25790, I25791, I25798, I25799, I25810, I25811, I25812, I2582, I2583, I2584, I2589, I259, Z951, Z955, Z9861
13. Cystic fibrosis: E840, E8419, E848, E849
14. Disseminated intravascular coagulation: D65, O45021, O45022, O45023, O45029
15. Drug use disorder: F11*, F12*, F13*, F14*, F15*, F16*, F18*, F19*, Z715*
16. Dyslipidemia: E780, E7800, E7801, E781, E782, E783, E784, E7849, E785
17. Esophageal varices, bleeding: I8501, I8511
18. Esophageal varices, non-bleeding: I8510, I8500

19. Gastrointestinal bleeding: I8501, I8501, I8511, I8511, K2081, K2081, K2091, K2091, K2101, K2101, K2211, K2901, K2901, K2921, K2921, K2921, K2931, K2931, K2941, K2941, K2951, K2951, K2961, K2961, K2971, K2971, K2981, K2981, K2991, K2991, K31811, K31811, K5701, K5701, K5701, K5711, K5711, K5713, K5713, K5721, K5721, K5721, K5731, K5731, K5733, K5733, K5741, K5741, K5741, K5751, K5751,

K5753, K5753, K5781, K5781, K5781, K5791, K5791, K5793, K5793, K50011, K50011, K50111, K50111, K50811, K50811, K50911, K50911, K51011, K51011, K51211, K51211, K51311, K51311, K51411, K51411, K51511, K51511, K51811, K51811, K51911, K51911, K226, K250, K252, K254, K256, K260, K262, K264, K266, K270, K272, K274, K276, K280, K282, K284, K286, K5521, K5521, K5521, K625, K9161, K9162, K91840, K91841, K922, K9401, K9411, K9421, K9431, K920, K921

20. Hepatic encephalopathy: K7291, K7201, K7211, K7290
21. Hepatic failure unspecific: K7290, K7291, K7291
22. Hepatorenal syndrome: K767
23. Hypertension: I10*, I11*, I12*, I13*, I15*
24. Intracranial hemorrhage: I6000, I6001, I6002, I6010, I6011, I6012, I602, I6020, I6021, I6022, I6030, I6031, I6032, I604, I6050, I6051, I6052, I606, I607, I608, I609, I610, I611, I612, I613, I614, I615, I616, I618, I619, I6200, I6201, I6202, I6203, I621, I629, S06340A, S06341A, S06342A, S06343A, S06344A, S06345A, S06346A, S06347A, S06348A, S06349A, S06350A, S06351A, S06352A, S06353A, S06355A, S06356A, S06357A, S06358A, S06359A, S06360A, S06361A, S06362A, S06363A, S06364A, S06365A, S06366A, S06367A, S06368A, S06369A, S06370A, S06371A, S06372A, S06373A, S06374A, S06375A, S06376A, S06377A, S06378A, S06379A, S06380A, S06381A, S06382A, S06383A, S06384A, S06385A, S06386A, S06387A, S06388A, S06389A, S064X0A, S064X1A, S064X2A, S064X3A, S064X4A, S064X5A, S064X6A, S064X7A, S064X8A, S064X9A, S065X0A, S065X1A, S065X2A, S065X3A, S065X4A, S065X5A, S065X6A, S065X7A, S065X8A, S065X9A, S066X0A, S066X1A, S066X2A, S066X3A, S066X4A, S066X5A, S066X6A, S066X7A, S066X8A, S066X9A
25. Liver cirrhosis: K717, K7030, K7031, K7460, K7469
26. Liver transplant: T8640, T8641, T8642, T8643, T8649, Z4823, Z944
27. Obesity: E66*
28. Portal hypertension: K766
29. Sepsis or infection: A000, A001, A009, A0100, A0101, A0102, A0103, A0104, A0105, A0109, A011, A012, A013, A014, A020, A021, A0220, A0221, A0222, A0223, A0224, A0225, A0229, A028, A029, A030, A031, A032, A033, A038, A039, A040, A041, A042, A043, A044, A045, A046, A047, A0471, A0472, A048, A049, A050, A051, A052, A053, A054, A055, A058, A059, A060, A061, A062, A063, A064, A065, A066, A067, A0681, A0682, A0689, A069, A070, A071, A072, A073, A074, A078, A079, A080, A0811, A0819, A082, A0831, A0832, A0839, A084, A088, A09, A150, A154, A155, A156, A157, A158, A159, A170, A171, A1781, A1782, A1783, A1789, A179, A1801, A1802, A1803, A1809, A1810, A1811, A1812, A1813, A1814, A1815, A1816, A1817, A1818, A182, A1831, A1832, A1839, A184, A1850, A1851, A1852, A1853, A1854, A1859, A186, A187, A1881, A1882, A1883, A1884, A1885, A1889, A190, A191, A192, A198, A199, A200, A201, A202, A203, A207, A208, A209, A210, A211, A212, A213, A217, A218, A219, A220, A221, A222, A227, A228, A229, A230, A231, A232, A233, A238, A239, A240, A241, A242, A243, A249, A250, A251, A259, A260, A267, A268, A269, A270, A2781, A2789, A279, A280, A281, A282, A288, A289, A300, A301, A302, A303, A304, A305, A308, A309, A310, A311, A312, A318, A319, A320, A3211, A3212, A327, A3281, A3282, A3289, A329, A33, A34, A35, A360, A361, A362, A363, A3681, A3682, A3683, A3684, A3685, A3686, A3689, A369, A3700, A3701, A3710, A3711, A3780, A3781, A3790, A3791, A380, A381, A388, A389, A390, A391, A392, A393, A394, A3950, A3951, A3952, A3953, A3981, A3982,

A3983, A3984, A3989, A399, A400, A401, A403, A408, A409, A4101, A4102, A411, A412, A413, A414, A4150, A4151, A4152, A4153, A4159, A4181, A4189, A419, A420, A421, A422, A427, A4281, A4282, A4289, A429, A430, A431, A438, A439, A440, A441, A448, A449, A46, A480, A481, A482, A483, A484, A4851, A4852, A488, A4901, A4902, A491, A492, A493, A498, A499, A5001, A5002, A5003, A5004, A5005, A5006, A5007, A5008, A5009, A501, A502, A5030, A5031, A5032, A5039, A5040, A5041, A5042, A5043, A5044, A5045, A5049, A5051, A5052, A5053, A5054, A5055, A5056, A5057, A5059, A506, A507, A509, A510, A511, A512, A5131, A5132, A5139, A5141, A5142, A5143, A5144, A5145, A5146, A5149, A515, A519, A5200, A5201, A5202, A5203, A5204, A5205, A5206, A5209, A5210, A5211, A5212, A5213, A5214, A5215, A5216, A5217, A5219, A522, A523, A5271, A5272, A5273, A5274, A5275, A5276, A5277, A5278, A5279, A528, A529, A530, A539, A5400, A5401, A5402, A5403, A5409, A541, A5421, A5422, A5423, A5424, A5429, A5430, A5431, A5432, A5433, A5439, A5440, A5441, A5442, A5443, A5449, A545, A546, A5481, A5482, A5483, A5484, A5485, A5486, A5489, A549, A55, A5600, A5601, A5602, A5609, A5611, A5619, A562, A563, A564, A568, A57, A58, A5900, A5901, A5902, A5903, A5909, A598, A599, A6000, A6001, A6002, A6003, A6004, A6009, A601, A609, A630, A638, A64, A65, A660, A661, A662, A663, A664, A665, A666, A667, A668, A669, A670, A671, A672, A673, A679, A680, A681, A689, A690, A691, A6920, A6921, A6922, A6923, A6929, A698, A699, A70, A710, A711, A719, A740, A7481, A7489, A749, A750, A751, A752, A753, A759, A770, A771, A772, A773, A7740, A7741, A7749, A778, A779, A78, A790, A791, A7981, A7989, A799, A800, A801, A802, A8030, A8039, A804, A809, A8100, A8101, A8109, A811, A812, A8181, A8182, A8183, A8189, A819, A820, A821, A829, A830, A831, A832, A833, A834, A835, A836, A838, A839, A840, A841, A848, A8481, A8489, A849, A850, A851, A852, A858, A86, A870, A871, A872, A878, A879, A880, A881, A888, A89, A90, A91, A920, A921, A922, A9230, A9231, A9232, A9239, A924, A925, A928, A929, A930, A931, A932, A938, A94, A950, A951, A959, A960, A961, A962, A968, A969, A980, A981, A982, A983, A984, A985, A988, A99, B000, B001, B002, B003, B004, B0050, B0051, B0052, B0053, B0059, B007, B0081, B0082, B0089, B009, B010, B0111, B0112, B012, B0181, B0189, B019, B020, B021, B0221, B0222, B0223, B0224, B0229, B0230, B0231, B0232, B0233, B0234, B0239, B027, B028, B029, B03, B04, B050, B051, B052, B053, B054, B0581, B0589, B059, B0600, B0601, B0602, B0609, B0681, B0682, B0689, B069, B070, B078, B079, B08010, B08011, B0802, B0803, B0804, B0809, B081, B0820, B0821, B0822, B083, B084, B085, B0860, B0861, B0862, B0869, B0870, B0871, B0872, B0879, B088, B09, B1001, B1009, B1081, B1082, B1089, B150, B159, B160, B161, B162, B169, B170, B1710, B1711, B172, B178, B179, B180, B181, B182, B188, B189, B190, B1910, B1911, B1920, B1921, B199, B20, B250, B251, B252, B258, B259, B260, B261, B262, B263, B2681, B2682, B2683, B2684, B2685, B2689, B269, B2700, B2701, B2702, B2709, B2710, B2711, B2712, B2719, B2780, B2781, B2782, B2789, B2790, B2791, B2792, B2799, B300, B301, B302, B303, B308, B309, B330, B331, B3320, B3321, B3322, B3323, B3324, B333, B334, B338, B340, B341, B342, B343, B344, B348, B349, B350, B351, B352, B353, B354, B355, B356, B358, B359, B360, B361, B362, B363, B368, B369, B370, B371, B372, B373, B3741, B3742, B3749, B375, B376, B377, B3781, B3782, B3783, B3784, B3789, B379, B380, B381, B382, B383, B384, B387, B3881, B3889, B389, B390, B391, B392, B393, B394, B395, B399,

B400, B401, B402, B403, B407, B4081, B4089, B409, B410, B417, B418, B419, B420, B421, B427, B4281, B4282, B4289, B429, B430, B431, B432, B438, B439, B440, B441, B442, B447, B4481, B4489, B449, B450, B451, B452, B453, B457, B458, B459, B460, B461, B462, B463, B464, B465, B468, B469, B470, B471, B479, B480, B481, B482, B483, B484, B488, B49, B500, B508, B509, B510, B518, B519, B520, B528, B529, B530, B531, B538, B54, B550, B551, B552, B559, B560, B561, B569, B570, B571, B572, B5730, B5731, B5732, B5739, B5740, B5741, B5742, B5749, B575, B5800, B5801, B5809, B581, B582, B583, B5881, B5882, B5883, B5889, B589, B59, B600, B6000, B6001, B6002, B6003, B6009, B6010, B6011, B6012, B6013, B6019, B602, B608, B64, B650, B651, B652, B653, B658, B659, B660, B661, B662, B663, B664, B665, B668, B669, B670, B671, B672, B6731, B6732, B6739, B674, B675, B6761, B6769, B677, B678, B6790, B6799, B680, B681, B689, B690, B691, B6981, B6989, B699, B700, B701, B710, B711, B718, B719, B72, B7300, B7301, B7302, B7309, B731, B740, B741, B742, B743, B744, B748, B749, B75, B760, B761, B768, B769, B770, B7781, B7789, B779, B780, B781, B787, B789, B79, B80, B810, B811, B812, B813, B814, B818, B820, B829, B830, B831, B832, B833, B834, B838, B839, B850, B851, B852, B853, B854, B86, B870, B871, B872, B873, B874, B8781, B8782, B8789, B879, B880, B881, B882, B883, B888, B889, B89, B900, B901, B902, B908, B909, B91, B92, B940, B941, B942, B948, B949, B950, B951, B952, B953, B954, B955, B9561, B9562, B957, B958, B960, B961, B9620, B9621, B9622, B9623, B9629, B963, B964, B965, B966, B967, B9681, B9682, B9689, B970, B9710, B9711, B9712, B9719, B9721, B9729, B9730, B9731, B9732, B9733, B9734, B9735, B9739, B974, B975, B976, B977, B9781, B9789, B998, B999, R6521, R7881, I76, R6520, I00, I029, K9081, G000, G001, G002, G003, G008, G009, G01, G02, G042, I301, I330, I400, J1082, J120, J121, J122, J123, J1281, J1289, J129, J13, J14, J150, J151, J1520, J15211, J15212, J1529, J153, J154, J155, J156, J157, J158, J159, J160, J168, J17, J180, J181, J188, J189, J851, J852, J09X1, J09X2, J09X3, J09X9, J1000, J1001, J1008, J101, J102, J1089, J1100, J1108, J111, J112, J1181, J1182, J1183, J1189, J36, J200, J201, J202, J203, J204, J205, J206, J207, J208, J210, J211, J218, J219, A360, A361, A362, J00, J020, J028, J0300, J0301, J069, J440, J470, J340, J390, J391, K610, K611, K612, K613, K614, K630, K650, K651, K652, N10, N151, N3000, N3001, N340, N764, L0100, L0101, L0102, L0103, L0109, L011, L0201, L0202, L0203, L0211, L0212, L0213, L02211, L02212, L02213, L02214, L02215, L02216, L02219, L02221, L02222, L02223, L02224, L02225, L02226, L02229, L02231, L02232, L02233, L02234, L02235, L02236, L02239, L0231, L0232, L0233, L02411, L02412, L02413, L02414, L02415, L02416, L02419, L02421, L02422, L02423, L02424, L02425, L02426, L02429, L02431, L02432, L02433, L02434, L02435, L02436, L02439, L02511, L02512, L02519, L02521, L02522, L02529, L02531, L02532, L02539, L02611, L02612, L02619, L02621, L02622, L02629, L02631, L02632, L02639, L02811, L02818, L02821, L02828, L02831, L02838, L0291, L0292, L0293, L03011, L03012, L03019, L03021, L03022, L03029, L03031, L03032, L03039, L03041, L03042, L03049, L03111, L03112, L03113, L03114, L03115, L03116, L03119, L03121, L03122, L03123, L03124, L03125, L03126, L03129, L03211, L03212, L03213, L03221, L03222, L03311, L03312, L03313, L03314, L03315, L03316, L03317, L03319, L03321, L03322, L03323, L03324, L03325, L03326, L03327, L03329, L03811, L03818, L03891, L03898, L0390, L0391, L0889, L089, M0000, M00011, M00012, M00019, M00021, M00022, M00029, M00031, M00032, M00039, M00041, M00042, M00049, M00051, M00052, M00059,

M00061, M00062, M00069, M00071, M00072, M00079, M0008, M0009, M0010, M00111, M00112, M00119, M00121, M00122, M00129, M00131, M00132, M00139, M00141, M00142, M00149, M00151, M00152, M00159, M00161, M00162, M00169, M00171, M00172, M00179, M0018, M0019, M0020, M00211, M00212, M00219, M00221, M00222, M00229, M00231, M00232, M00239, M00241, M00242, M00249, M00251, M00252, M00259, M00261, M00262, M00269, M00271, M00272, M00279, M0028, M0029, M0080, M00811, M00812, M00819, M00821, M00822, M00829, M00831, M00832, M00839, M00841, M00842, M00849, M00851, M00852, M00859, M00861, M00862, M00869, M00871, M00872, M00879, M0088, M0089, M009, M01X0, M01X11, M01X12, M01X19, M01X21, M01X22, M01X29, M01X31, M01X32, M01X39, M01X41, M01X42, M01X49, M01X51, M01X52, M01X59, M01X61, M01X62, M01X69, M01X71, M01X72, M01X79, M01X8, M01X9, M4620, M4621, M4622, M4623, M4624, M4625, M4626, M4627, M4628, M8600, M86011, M86012, M86019, M86021, M86022, M86029, M86031, M86032, M86039, M86041, M86042, M86049, M86051, M86052, M86059, M86061, M86062, M86069, M86071, M86072, M86079, M8608, M8609, M8610, M86111, M86112, M86119, M86121, M86122, M86129, M86131, M86132, M86139, M86141, M86142, M86149, M86151, M86152, M86159, M86161, M86162, M86169, M86171, M86172, M86179, M8618, M8619, T80211A, T80211D, T80211S, T80212A, T80212D, T80212S, T80218A, T80218D, T80218S, T80219A, T80219D, T80219S, T8022XA, T8022XD, T8022XS, T8029XA, T8029XD, T8029XS, T8140XA, T8140XD, T8140XS, T8144XA, T8144XD, T8144XS.

30. Spontaneous bacterial peritonitis: K652
31. Tobacco use disorder: Z720*, Z87891, O9933*, F17*
32. Unspecified hepatic failure: K7290, K7291, K7291
33. Venous thromboembolism: I81, I820, I82210, I82211, I82220, I82221, I82290, I82291, I823, I82401, I82402, I82403, I82409, I82411, I82412, I82413, I82419, I82421, I82422, I82423, I82429, I82431, I82432, I82433, I82439, I82441, I82442, I82443, I82449, I82491, I82492, I82493, I82499, I824Y1, I824Y2, I824Y3, I824Y9, I824Z1, I824Z2, I824Z3, I824Z9, I82501, I82502, I82503, I82509, I82511, I82512, I82513, I82519, I82521, I82522, I82523, I82529, I82531, I82532, I82533, I82539, I82541, I82542, I82543, I82549, I82591, I82592, I82593, I82599, I825Y1, I825Y2, I825Y3, I825Y9, I825Z1, I825Z2, I825Z3, I825Z9, I82621, I82622, I82623, I82629, I82721, I82722, I82723, I82729, I82A11, I82A12, I82A13, I82A19, I82A21, I82A22, I82A23, I82A29, I82B11, I82B12, I82B13, I82B19, I82B21, I82B22, I82B23, I82B29, I82C11, I82C12, I82C13, I82C19, I82C21, I82C22, I82C23, I82C29, I2602, I2609, I2692, I2699, I2782, Z86711.

B: Procedure codes {International Classification of Diseases, Tenth Revision Procedure Coding System (ICD-10-PCS)}

1. Liver transplant as procedure: 0FY00Z0, 0FY00Z1, 0FY00Z2
2. Invasive mechanical ventilation: 5A1935Z, 5A1945Z, 5A1955Z, 0BH17EZ, 0BH18EZ
3. Non-invasive ventilation: 5A09357, 5A09457, 5A09557, 5A09358, 5A09458, 5A09558

C: Median household income values

Table S1. The quartile classification of the estimated median household income of residents in the patient’s ZIP code vary by every year. Listed below are the dollar ranges for study duration 2017-2019.

**Year**	**Quartile 1**	**Quartile 2**	**Quartile 3**	**Quartile 4**
2016	1 - 42,999	43,000 - 53,999	54,000 - 70,999	71,000+
2017	1 - 43,999	44,000 - 55,999	56,000 - 73,999	74,000+
2018	1 - 45,999	46,000 - 58,999	59,000 - 78,999	79,000+
2019	1 - 47,999	48,000 - 60,999	61,000 - 81,999	82,000+
2020	1 - 49,999	50,000 - 64,999	65,000 - 85,999	86,000+
